# Fifty years on: Serotonin and depression

**DOI:** 10.1177/02698811231161813

**Published:** 2023-03-20

**Authors:** Sameer Jauhar, Philip J Cowen, Michael Browning

**Affiliations:** 1 Department of Psychological Medicine, IoPPN, King’s College, London, UK; 2 Department of Psychiatry, University of Oxford, Warneford Hospital Oxford, Oxon, UK

**Keywords:** Serotonin, depression, psychopharmacology, learning, positron emission tomography

## Abstract

It has been over 50 years since the original serotonin hypothesis was proposed by the British Psychiatrist Alec Coppen. Recently, some authors have questioned the validity of the hypothesis. In this narrative review, we summarise the evidence for the serotonin hypothesis of depression, focusing on psychopharmacology and molecular imaging, as well as systems-level neuroscience.

The notion that clinical depression might be caused by deficient activity of the brain neurotransmitter, serotonin (5-hydroxytryptamine (5-HT)) is over 50 years old. This theory seems to have been first proposed in 1967 by the British psychiatrist, Alec Coppen, though in his review, Coppen mentions several other lines of aetiological research, including the potential roles for noradrenaline, excess cortisol secretion and electrolyte disturbances (Coppen, 1967).

Direct investigation of neurochemistry in the living human brain was not possible when Coppen was writing, and a substantial part of the evidence supporting the serotonin hypothesis was derived from the actions of antidepressant drugs, such as monoamine oxidase inhibitors and tricyclic antidepressants, which had recently been shown in animal experimental work to potentiate the action of serotonin at neuronal synapses. Coppen, however, rightly cautioned that ‘the actions of these drugs may merely represent therapeutic manoeuvres which in themselves may be quite unrelated to aetiological factors underlying the majority of cases of depression’.

In recent years, while selective modification of serotonin neurotransmission has continued to be a therapeutic option in the treatment of mood and anxiety disorders, and the efficacy of the selective serotonin reuptake inhibitors (SSRIs) for a significant number of people (though not all) has been demonstrated (Cipriani et al., 2018; Hieronymus et al., 2020), the notion that a complex heterogeneous conditions such as clinical depression could be caused by deficient functioning of a single neurotransmitter has been regarded as implausible (Cowen and Browning, 2015).

Current theories are usually based on systems-level neuroscience and implicate circuitry involved in key neurobiological domains, such as emotional processing, reward/reinforcement learning and decision-making. While there has continued to be intense research interest into the role of serotonin in neuropsychological functioning and the actions of pharmacological agents, particularly psychedelics, the serotonin hypothesis of depression seemed to be enjoying a well-earned retirement.

Nevertheless, last July, to the surprise of many, the role of serotonin in depression was trending on multiple media outlets, as well as PubMed. The reason for this was a paper by Joanna Moncrieff and colleagues, entitled ‘The serotonin theory of depression: a systematic umbrella review of the evidence’ (Moncrieff et al., 2022). That such an unpromisingly titled academic paper could create widespread public interest vindicates the view of Moncrieff et al. that the serotonin hypothesis retains a considerable reach on how people understand the causation of depression (though the 2011 Australian survey cited by Moncrieff et al. showed more people attributed depression to life events and psychosocial stressors than a ‘chemical imbalance’; Pilkington et al., 2013).

However, perhaps the main target of the paper (and the reason for it capturing the public space) was the putative relationship between the serotonin hypothesis of depression and prescribing of SSRIs. The press release from University College, London commented: ‘SSRIs. . . were originally said to work by correcting abnormally low serotonin levels. There is no other pharmacological mechanism by which antidepressants affect the symptoms of depression’. Alec Coppen’s warning had apparently gone unheeded.

The abstract of the paper by Moncrieff et al. (2022) stated there was ‘no support for the hypothesis that depression is caused by lowered serotonin activity or concentrations.’ In fact, this conclusion is arguable and is not consistent with the data reviewed by Moncrieff et al. The aim of the current narrative review is to briefly summarise the more reliable abnormalities in serotonin mechanisms found in (ideally) unmedicated depressed patients and relate these to the integrated models of depression found in current neurobiological formulations of aetiology.

## Plasma tryptophan in depression

Serotonin is synthesised from a precursor amino acid, tryptophan. Tryptophan is an essential amino acid which cannot be produced in the body and its availability to the brain from blood is a rate-limiting step in the production of brain serotonin. Sampling peripheral plasma tryptophan in humans is straightforward and three meta-analyses have concluded that plasma tryptophan is decreased in unmedicated depressed patients, with effect sizes increasing from Hedge’s *g* = 0.45 in all patients to 0.84 in unmedicated patients (Ogawa et al., 2014), a subsequent meta-analysis finding similar effect sizes in major depressive disorder (MDD) patients (Standardised Mean Difference (SMD) = −0.46) (Pu et al., 2021), and recent replication (SMD = −0.51) (Almulla et al., 2022).

Neither the role that diminished plasma tryptophan might play in the causation of depression nor the mechanism that underlies this abnormality is well understood. Current theories have implicated a role for inflammation and the induction of tryptophan metabolising enzymes such as indolamine 2,3-dioxygenase. This could lead to increased metabolism of tryptophan through the kynurenine pathway, potentially giving rise to neurotoxic metabolites such as quinolinic acid. However, whether the kynurenine pathway is activated in patients with major depression is open to question (Almulla et al., 2022; Marx et al., 2021).

### Tryptophan depletion

The critical role that tryptophan availability plays in brain serotonin synthesis has led to some ingenious techniques designed to lower brain serotonin levels acutely through dietary manipulation. The most widely used technique is ‘tryptophan depletion’ where administration of an amino acid load which lacks tryptophan lowers plasma tryptophan levels by about 80% over a few hours (Carpenter et al., 1998). In both animals (Biggio et al., 1974) and humans (Concu et al., 1977), this produces a significant decrease in brain serotonin synthesis, though the exact mechanism remains unclear. Nonetheless, tryptophan depletion offers a straightforward way to test the serotonin hypothesis of depression.

It is well established that tryptophan depletion in healthy participants, who lack obvious risk factors for depression, does not cause significant lowering of mood (Ruhé et al., 2007). Therefore, the notion that decreasing brain serotonin levels is sufficient to cause depression can be rejected. However, in people recovered from depression and free of pharmacological and psychological treatment for long periods of time, tryptophan depletion produces a clinically significant lowering of mood. Such an effect is apparent in the review by Moncrieff et al. (2022) and was reported in a highly cited meta-analysis, cited in the aforementioned ‘umbrella’ review (Ruhé et al., 2007). The effect size for mood symptoms in remitted antidepressant free depression patients was 1.9, from 8 samples, with removal of a potential outlier giving Hedge’s *g* = −1.06.

From this, it seems reasonable to conclude that diminished serotonin levels in the brain are neither necessary nor sufficient to cause clinical depression. However, in those at risk of depression through suffering previous episodes, reductions in brain serotonin levels can lead to clinical relapse. Presumably here, decreased activity of serotonin pathways interacts with important pre-existing neurobiological vulnerabilities, probably in the regulation of key neural networks (Fusar-Poli et al., 2006). The widespread neuromodulatory role of serotonin is likely to be relevant to this (see below).

### Serotonin-mediated neuroendocrine responses

Prior to the advent of brain imaging, serotonin-mediated neuroendocrine responses were employed as a means of assessing the function of brain serotonin neurons involved in anterior pituitary hormone release. The most consistent data in depressed patients are from pharmacological challenges that increase serotonin activity and plasma prolactin levels by occupying the serotonin transporter on serotonin nerve terminals. Two agents have been employed for this purpose, clomipramine and citalopram. The literature reveals four studies, two with clomipramine and two with citalopram; all found a decreased prolactin response in unmedicated depressed patients (Anderson et al., 1992; Bhagwagar et al., 2002; Golden et al., 1992; Kapitany et al., 1999). Similar findings were reported in a small study of depressed adolescents (Sallee et al., 1998). It is possible that impaired prolactin release to challenge with serotonin reuptake inhibitors is a trait marker of vulnerability to depression because the abnormality apparently persists in patients recovered from depression and withdrawn from antidepressant medication (Bhagwagar et al, 2002).

### Serotonin imaging in the brain

Positron emission tomography (PET) and single photon emission tomography (SPET) have allowed more direct assessment of serotonergic mechanisms in the human brain; however, the technical difficulty and expense of this work have limited the number of studies.

The most investigated serotonin receptors in depressed patients are the 5-HT_1A_ receptor and the serotonin transporter.

5-HT_1A_ receptors are divided into two classes, dependent on location. First, they are located on soma and dendrites of serotonin neurons in the raphe nuclei in the brainstem, where they act as an inhibitory autoreceptor (Yohn et al., 2017). Second, large numbers of 5-HT_1A_ receptors are also found post-synaptically (as heteroreceptors) in cortical and limbic regions.

Most relevant PET studies have included depressed patients who were either drug naïve or drug free for long periods of time. The majority of studies have reported decreased 5-HT_1A_ receptor binding. A meta-analysis cited by Moncrieff et al. (Wang et al., 2016) included 10 studies, half of which included mixed populations of people with bipolar disorder or postpartum depression. Most of these studies used BP_ND_, which only measures brain activity of ligand, and therefore does not require arterial blood sampling, measuring difference between target and reference region, assuming negligible activity in the reference region.

All but one study (in postpartum depression) included people who were drug free.

The meta-analysis reported a large reduction in 5-HT_1A_ receptors in mesiotemporal cortex with smaller decreases in other post-synaptic areas and the raphe nuclei (Wang et al., 2016).

A problem with interpreting this literature has been the replicated finding by one group of *increased* 5-HT_1A_ receptor binding in unmedicated depressed patients across all brain regions (Kaufman et al., 2016). Specifically, Parsey et al. showed higher binding with an arterial input function (obviating need for a reference region) and, using similar methods to the studies cited in the aforementioned meta-analysis, lowered binding when grey matter cerebellum was used as a reference region. Their explanation was that binding in grey matter in patients compared to controls would account for these discrepant findings (Parsey et al., 2010). Other studies in the meta-analysis that used white matter cerebellum as reference region were in people with bipolar depression, with discrepant findings, and therefore drawing conclusions is difficult. Binding could reflect a number of different causes, such as changes in receptor density or affinity.

The advent of new PET ligands (most probably agonists; Selvaraj et al., 2012) for the 5-HT_1A_ receptor may enable resolution of this issue. Until then, a degree of agnosticism seems appropriate concerning the status of brain 5-HT_1A_ receptors in depressed patients revealed by PET.

#### Serotonin transporters

In PET and SPET studies, the highest density of serotonin transporters is found in the brainstem and midbrain, where serotonin cell bodies are concentrated. Meta-analyses have shown reliable reductions in transporter binding in these brain regions as well as in the amygdala (Gryglewski et al., 2014; Kambeitz and Howes, 2015), the latter study also shows reductions in striatum. Decreases in transporter binding are seen in patients unmedicated for long periods of time and also those who are antidepressant naïve (Yeh et al., 2015). The mechanism that underlies these reductions in serotonin transporter binding is not established, which may represent decreased activity or decreased numbers of serotonin neurons in people with depression.

PET studies have potential to image neurotransmitter release in vivo, involving an appropriate pharmacological challenge to release endogenous neurotransmitter followed by measurement of the amount of ligand displaced from post-synaptic receptors. This approach has been used with success in the investigation of psychosis to show that acute psychotic symptoms are associated with increased dopamine release with amphetamine challenge in people with schizophrenia (Laruelle and Abi-Dargham, 1999), dopamine depletion agents revealing increased receptor binding, indicating less endogenous dopamine (Abi-Dargham et al., 2000). However, it has proved challenging to identify a serotonin receptor ligand that is readily displaceable by endogenous serotonin. Pharmacological manipulation studies have historically failed to demonstrate in vivo change in ligand binding for the 5 HT_1A_ receptor and SERT following tryptophan depletion (Praschak-Rieder et al., 2004; Talbot et al., 2005), and a [(^11^)C]WAY-100635 PET study examining 5 HT_1A_ receptor binding with tryptophan infusion similarly failed to detect an effect (Rabiner et al., 2002) (for detailed review, see Patterson et al., 2010).

Recently, however, it has been demonstrated that d-amphetamine administration produces sufficient serotonin release to displace the 5-HT_2A_ receptor ligand, [^11^C]Cimbi-36 from frontal cortex, and a study using this technique found diminished serotonin release in 17 unmedicated depressed patients. (Erritzoe et al., 2022). However, the sample included a number of people with Parkinson’s disease, and the control group was not matched for age (though the latter should not theoretically impact findings); though the findings were rightfully acknowledged as important, though preliminary.

This study requires replication but demonstrates that as techniques advance it should be possible to assess the activity of serotonin neurons in depression with greater precision.

### Status of serotonin activity in depression

The evidence summarised here shows that there are some reliable abnormalities in serotonin activity in unmedicated depressed patients. Currently, a simple synthesis of these abnormalities is elusive, but overall the evidence suggests a decrease in the activity of presynaptic serotonin neurons. This would be consistent with diminished tryptophan availability to the brain, impaired serotonin-mediated endocrine responses to presynaptic challenge, and lowered serotonin transporter binding on raphe cell bodies in the brainstem. Decreased serotonin release in the brain in response to amphetamine challenge, if replicated, would further support this view.

While these changes in the serotonin activity in depressed patients are of interest, they do not necessarily represent causal mechanisms. However, the work with tryptophan depletion suggests that in some circumstances, particularly in patients who have experienced recurrent depression, low serotonin activity could be involved in predisposing to clinical relapse. This might be relevant to the role of maintenance antidepressant treatment in people at high risk of recurrent illness.

### Systems-level role of serotonin in cognition

When assessing whether the serotonergic system is involved in the aetiology of depression, it is useful to first consider the role serotonin plays in how the brain processes information. In common with other neuromodulatory neurotransmitters, there are relatively few serotonergic neurons in the brain, their nuclei are concentrated in a small region of the brainstem, and they project widely throughout both the cortex and sub-cortical structures (Muller and Jacobs, 2009). This anatomical arrangement is well suited to the transmission of relatively simple, globally relevant messages across multiple regions of the brain. While the complexity of the serotonergic system is increased by a broad range of functionally distinct receptors and anatomically specific subnuclei projections (Muller and Jacobs, 2009), there has been continued interest in determining what the content of these messages might be. The paradigmatic example of this approach has examined dopaminergic neurons originating in the ventral tegmental area. The activity of approximately 50% of these dopaminergic neurons is well described by ‘reward prediction error’ signals (Schultz et al., 1997), which carry the message ‘that was better than you thought it was going to be’ (Sutton and Barto, 2018).

To date, the message(s) conveyed by the serotonergic system have not been as convincingly characterised as they have for the dopaminergic system. Candidate serotonergic messages draw on the behavioural effect of serotonergic stimulation in experimental settings, which tends to lead to the withholding or inhibition of behaviour, and the observation that serotonergic neurons seem to be activated by both punishing and rewarding events (Dayan and Huys, 2008; Miyazaki et al., 2011). Thus, it has been suggested that serotonin carriers a ‘punishment prediction error’ signal (‘that was worse than you thought it was going to be’) (Daw et al., 2002), or possibly an estimate of the expected rate at which adversity will be encountered (‘It is risky to act at the moment’) (Cools et al., 2011).

A second variant of message linked to serotonin function concerns how future events are evaluated and seeks to explain why increasing levels of serotonin cause a reduction in impulsivity. As a rule, rewards that are immediately available are preferred over those which are delayed and serotonin has been argued to reduce ‘delay-discounting’, the rate at which time erodes the value of rewarding events (‘it is worth waiting’) (Miyazaki et al., 2014; Schweighofer et al., 2008). Lastly, an intriguing suggestion frames the evaluative and prospective dimensions of the serotonergic message in terms of trains of thought. Specifically, it is suggested that when deciding what action to take it is useful to imagine both the immediate outcome of your actions and the outcomes of the subsequent actions that then become available. This process is akin to following the branches of a tree from the trunk to the tips, with each branch representing a series of sequential actions. In many cases, the number of future outcomes to be considered becomes extremely large very quickly (i.e. the tree often has many branches that divide often) making this a challenging task. Serotonin is argued to simplify the problem by halting the search along a specific branch as soon as an unwanted outcome is reached, in effect pruning the tree (‘don’t go there!’) (Huys et al., 2012).

None of the proposed serotonergic messages enjoy the broad, cross-species empirical support of the dopaminergic reward prediction-error account and should therefore be considered as pointers to serotonin’s overall role in cognitive function rather than definitive descriptions. However, a common factor across all of the potential serotonergic messages is that they contain information about the estimated value of events, a quantity which, when measured, is disordered in depressed patients (Huys and Browning, 2022) and is a core component of cognitive accounts of the illness. This observation clearly does not provide compelling evidence that disordered serotonergic function is a significant aetiological factor in the development or maintenance of depression; however, it does suggest an intermediate cognitive mechanism by which altering serotonergic function (e.g. via tryptophan depletion, treatment with serotonergic medication or pathology) might lead to changes in depressive symptoms.

## Conclusion

What should we make of Alec Coppen’s insight, over 50 years on? Serotonergic agents continue to be widely employed in the treatment of a range of mental health conditions, particularly anxiety and depression. Indeed, one of the more dramatic demonstrations of the role of serotonin in mood and self-conscious experience comes from the study of psychedelic drugs, now being repurposed for the treatment of resistant depression.

As we have described, there are some reliable abnormalities in serotonin mechanisms in depressed patients but their potential role in the causation of illness remains to be determined. A likely aid in resolving this question will be the continued intense interest in pre-clinical studies of the role of serotonin in processes relevant to depression such as reward and punishment learning, decision-making, emotional regulation, and social cognition (Roberts et al., 2020). Along with this, in clinical studies, there will be improvements in methods of assessing brain serotonin activity, as shown by recent investigations measuring serotonin release in the living human brain. Clearly, the role of serotonin in depression will need to be integrated into more complex neurobiological models than those originally envisaged. Nevertheless, the link between impaired serotonin activity and depression is likely to outlive its recent obituaries.
